# Adaptive Model Predictive Control for 4WD-4WS Mobile Robot: A Multivariate Gaussian Mixture Model-Ant Colony Optimization for Robust Trajectory Tracking and Obstacle Avoidance

**DOI:** 10.3390/s25123805

**Published:** 2025-06-18

**Authors:** Hayat Ait Dahmad, Hassan Ayad, Alfonso García Cerezo, Hajar Mousannif

**Affiliations:** 1 Laboratory of Electrical Systems, Energy Efficiency and Telecommunications, Faculty of Science and Technics, Cadi Ayyad University (UCA), Marrakech 40000, Morocco; h.ayad@uca.ma; 2 Department of Systems Engineering and Automation, University of Malaga, 29016 Malaga, Spain; gcerezo@ctima.uma.es; 3 Faculty of Science Semlalia, Cadi Ayyad University (UCA), Marrakech 40000, Morocco; mousannif@uca.ac.ma

**Keywords:** trajectory tracking, MPC, MGMM-ACO*_R_* algorithm, 4WD-4WS mobile robot

## Abstract

Trajectory tracking is a crucial task for autonomous mobile robots, requiring smooth and safe execution in dynamic environments. This study uses a nonlinear model predictive controller (MPC) to ensure accurate trajectory tracking of a four-wheel drive, four-wheel steer (4WD-4WS) mobile robot. However, the MPC’s performance depends on the optimal tuning of its key parameters, a challenge addressed using the Multivariate Gaussian Mixture Model Continuous Ant Colony Optimization (MGMM-${\mathrm{ACO}}_{R}$) algorithm. This method improves on the classic ${\mathrm{ACO}}_{R}$ algorithm by overcoming two major limitations: the lack of consideration for interdependencies between optimized variables, and an inadequate balance between global exploration and local exploitation. The proposed approach is validated by a two-phase evaluation. Firstly, benchmark function evaluations demonstrate its superiority over established optimization algorithms, including ACO, ${\mathrm{ACO}}_{R}$, and PSO and its variants, in terms of convergence speed and solution accuracy. Secondly, MGMM-${\mathrm{ACO}}_{R}$ is integrated into the MPC framework and tested in various scenarios, including trajectory tracking with circular and eight trajectories and dynamic obstacle avoidance during trajectory tracking. The results, evaluated based on trajectory error, control effort, and computational latency, confirm the robustness of the proposed method. In particular, the explicit modeling of correlations between variables in MGMM-${\mathrm{ACO}}_{R}$ guarantees stable, collision-free trajectory tracking, outperforming conventional ${\mathrm{ACO}}_{R}$-based approaches that optimize variables independently.

## 1. Introduction

In recent years, robotics has made significant advances, with applications spanning diverse fields such as space exploration, aerospace, and agriculture [1,2,3]. These developments have driven researchers to refine control methods for robotic systems, aiming to enhance their accuracy, efficiency, and safety in performing tasks. The growing complexity of robotic missions, whether navigating difficult extraterrestrial terrain [4], optimizing crop management [5], or maintaining aeronautical systems, highlights the need for innovative control techniques.

A wide variety of robotic platforms, including mobile robots, unicycles, four-wheel drive and four-wheel steer robots, robotic arms, drones, and more, are used to accomplish these tasks. Despite the diversity in their designs and functionalities, these robots face a common challenge: motion control. This aspect is crucial, as effective motion control enables robots to navigate accurately, efficiently, and reliably to achieve their intended objectives, particularly in dynamic and unpredictable environments. Addressing this challenge has prompted the development of advanced solutions, which include both innovative and classical algorithms, as well as various control strategies.

In terms of classical approaches, the author of [6] has designed a PID controller integrated with a Kalman filter to attenuate noise during robotic system operation, which is often affected by phenomena such as slippage and vibration. This study demonstrated the effectiveness of the Kalman filter when combined with a PID controller, underlining its value in improving system performance. Similarly, in [7], a variable-parameter PID controller was developed for a differential-drive mobile robot to follow a predefined trajectory with a desired time-varying velocity. The proposed method was validated through both simulations and experimental results, demonstrating its effectiveness in achieving precise trajectory tracking. To address more complex control challenges, a novel sliding-mode-based variable structure controller was introduced in [8] for trajectory tracking in wheeled mobile robots. This approach combined a sliding mode algorithm with the backstepping method, ensuring rapid and stable convergence of trajectory-tracking errors to zero.

Regarding advanced methods or intelligent controllers, fuzzy logic controllers have been extensively studied for their ability to emulate human reasoning in robotic systems. In [9], the authors presented a fuzzy logic-based approach to guide a mobile robot toward a predefined destination using GPS data. Formulating expert-designed rules enabled the robot to navigate with high precision, achieving minimal error. This approach has also been utilized for the navigation of agricultural robots. In [10], a type-2 fuzzy logic controller was developed to facilitate navigation in both obstacle-free and obstacle-occupied environments. A supervisory mechanism was implemented to seamlessly switch between controllers designed for each specific scenario, enabling the robot to navigate the environment safely and effectively. Additionally, fuzzy logic has demonstrated highly efficient results, even in dynamic, obstacle-occupied environments. In [11], a fuzzy logic controller was implemented within a multilayer decision framework, utilizing prediction and priority rules to enhance the quality of the robot’s next position. This approach took into account critical factors such as path length, safety, and runtime. The results of comparative studies revealed a considerable improvement.

In the context of intelligent controllers, while fuzzy logic has proven effective in handling uncertainty, researchers have recently turned to reinforcement learning (RL) techniques for environments where little to no prior information is available. This is particularly relevant in complex and unpredictable scenarios, such as space exploration, where RL enables systems to learn and adapt through interaction with the environment. A study [12] applied a deep reinforcement learning strategy to enable a mobile robot to navigate from an initial position to a specified destination. Specifically, the Deep Q-Learning algorithm was utilized, incorporating a federated learning approach to address privacy concerns and establish a solid foundation for secure and efficient navigation. In [13], a path-tracking trajectory approach based on reinforcement learning, specifically Q-learning, was developed. This method features real-time obstacle avoidance capabilities and is applied to control wheeled mobile robots in dynamic local environments. Simulation results confirm the effectiveness of the proposed approach in accomplishing the robot’s tasks. RL has also been investigated in the field of path-following and obstacle-avoidance control for nonholonomic wheeled mobile robots, demonstrating remarkable results in real-world experimental validation [14].

Building on advancements in control and navigation, model predictive control (MPC) has gained prominence as a practical and industrial-grade solution [15,16,17]. Unlike RL, which often requires extensive training and can be computationally intensive [18], MPC provides a more systematic approach that ensures stability and optimal performance in dynamic environments. Known for its ease of implementation, MPC uses an optimization-based framework to predict system behavior and compute real-time control actions. This makes MPC particularly suitable for applications where timely and efficient decision-making is critical. Several studies have been carried out using this controller, including developing an MPC controller in [19] for a four-wheeled omni robot. This approach enabled the robot to smoothly follow a specified trajectory while avoiding collisions with surrounding obstacles. Simulation results validated the effectiveness of the proposed method. In [20], a motion control approach was developed using an MPC controller for skid-steer mobile robots in agricultural scenarios. The proposed method demonstrated strong performance in both obstacle-free and obstacle-present environments, achieving a reduced tracking error.

The primary challenge in MPC lies in optimizing the trade-off between state and control matrices to balance tracking performance and control effort. Conventional trial-and-error tuning often compromises controller reliability and induces sub-optimal performance. Although RL algorithms can automate the adaptation of MPC parameters [21], their effectiveness depends on the prior optimization of specific RL hyperparameters, a computationally intensive metaoptimization problem. Poorly configured RL hyperparameters (e.g., learning rates, discount factors) risk destabilizing the system and require substantial computational resources for convergence. Crucially, this design challenge constitutes a constrained optimization problem. Metaheuristic algorithms [22,23,24] address this limitation by enabling computationally efficient off-line optimization of critical MPC parameters. Unlike RL, which requires exhaustive tuning of hyperparameters, metaheuristics achieve robust control performance with fewer algorithmic parameters (e.g., population size, mutation probability), considerably simplifying deployment while maintaining adaptability to constraints.

Building on the insights gained from the foregoing research, we propose a new optimization approach designed to improve the performance of model predictive control (MPC) by optimizing its weighting matrices. This method extends the principles of Continuous Ant Optimization (${\mathrm{ACO}}_{R}$) and introduces an advanced framework, called Continuous Ant Optimization based on a multivariate Gaussian Mixture Model, MGMM-${\mathrm{ACO}}_{R}$. MGMM-${\mathrm{ACO}}_{R}$ leverages the strengths of traditional ${\mathrm{ACO}}_{R}$ while incorporating a probabilistic multivariate Gaussian Mixture Model to improve exploration and exploitation capabilities in high-dimensional optimization problems. In addition, it accounts for correlations between variables and reduces the number of algorithm parameters compared with traditional ${\mathrm{ACO}}_{R}$. In so doing, this approach guarantees a more efficient and adaptive search process, which ultimately translates into improved MPC performance in terms of trajectory tracking and dynamic obstacle avoidance.

Our contributions in this work are summarized as follows:

- Novel Optimization Framework: We introduce the MGMM-${\mathrm{ACO}}_{R}$, an advanced optimization method that integrates multivariate Gaussian Mixture Models with Continuous Ant Colony Optimization to enhance the search process and to achieve superior performance in complex optimization tasks.
- Benchmark Testing and Comparative Analysis: The proposed MGMM-${\mathrm{ACO}}_{R}$ is rigorously tested using standard benchmark functions and its performance is compared against traditional Ant Colony Optimization (ACO) and Continuous Ant Colony Optimization (${\mathrm{ACO}}_{R}$) algorithms. The results demonstrate significant improvements in terms of convergence speed.
- MPC Weighting Matrix Optimization: We apply MGMM-${\mathrm{ACO}}_{R}$ to optimize the weighting matrices of MPC, enabling a more effective balance between path tracking accuracy and computational efficiency.
- Evaluation in Diverse Scenarios: The optimized MPC is evaluated in various scenarios, including path tracking and dynamic obstacle avoidance. The results highlight the effectiveness of the proposed method, ensuring smooth and safe trajectories.

## 2. Classical ACO*_R_* Algorithm

Continuous Ant Colony Optimization (${\mathrm{ACO}}_{R}$) is an optimization metaheuristic algorithm inspired by the foraging behavior of ants, first introduced by Socha [25]. This algorithm is an extension of the traditional Ant Colony Optimization (ACO), which was originally developed to solve combinatorial optimization problems. Unlike classical ACO, which relies on discrete probability distributions, ${\mathrm{ACO}}_{R}$ utilizes a continuous probability density function (PDF) to explore and identify promising regions within the search space. Solutions are sampled from this PDF, which is iteratively updated based on the best solutions discovered, allowing the algorithm to efficiently tackle continuous optimization problems.

**Figure 1:**
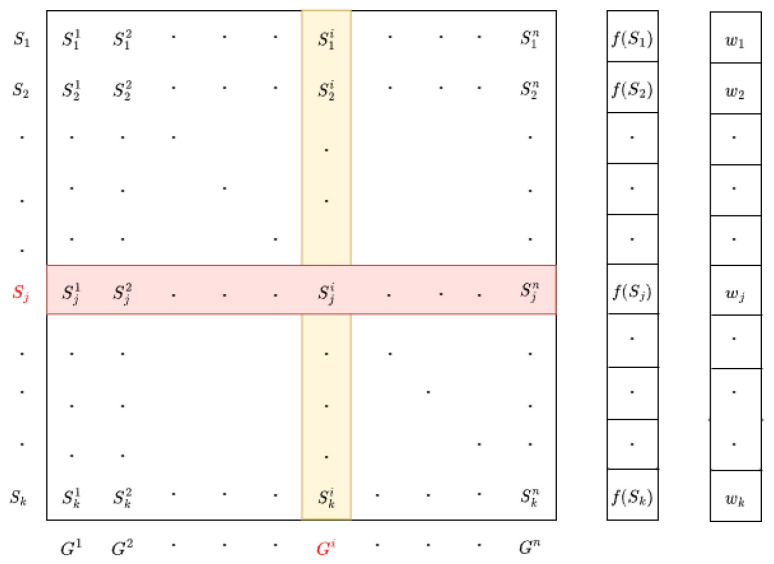
Population archive of ${\mathrm{ACO}}_{R}$ algorithm.

The ${\mathrm{ACO}}_{R}$ algorithm utilizes a population archive to track solutions, as depicted in Figure 1. This archive is a structured list that retains a fixed number k of high-quality solutions for the optimization problem. Each solution within the archive is characterized by n decision variables, corresponding to the dimensions of the problem. The archive is organized by sorting the solutions based on their fitness values f, with each solution assigned a specific weight. These weights are calculated using Equation (1).

(1) $$\omega_{j}=\frac{1}{qk\sqrt{2\pi }}e^{-\frac{{\left(rank\left(j\right)-1\right)}^{2}}{2q^{2}k^{2}}}$$

The equation defines the weight assigned to a solution $S_{j}$, where the parameters are interpreted as follows:

*k*: Represents the size of the solution archive, indicating the number of elite solutions retained at each iteration. It also determines the breadth of the weight distribution.
q: Denotes the selection pressure parameter, which controls the rate at which weights decay with increasing solution rank, thus balancing exploration and exploitation.
q*k*: Defines the standard deviation σ of the Gaussian function employed to assign weights.

After computing the weight of each solution using (1), a corresponding probability is assigned to each solution. The solution with the lowest fitness value, representing the optimal outcome, is assigned the highest probability. For every solution stored in the population archive, Gaussian sampling is then applied using the following equation:

(2) $$g\left(x,\mu ,\sigma \right)=\frac{1}{\sigma \sqrt{2\pi }}e^{-\frac{{\left(x-\mu \right)}^{2}}{2\sigma^{2}}}$$

μ is equal to $S_{j}^{i}$, while σ represents the average distance between the selected solution $S_{j}$ and all other solutions in the archive at construction step i. Consequently, σ is calculated using (3), where ξ is the deviation distance parameter of the algorithm.

(3) $$\sigma_{j}^{i}=\xi \sum_{m=1}^{k}\frac{\left|s_{m}^{i}-s_{j}^{i}\right|}{k-1}.$$

The process is repeated for each dimension, and the average distance $\sigma_{j}^{i}$ is recalculated at each iteration. In ${\mathrm{ACO}}_{R}$, a population archive is used to store solutions, and the algorithm incorporates a structured procedure to update this archive. The archive size, denoted as *k*, must be at least equal to the number of dimensions of the problem being addressed.

When the algorithm is initialized, *k* solutions are generated and stored in the population archive. Once this initial population has been established, the update phase begins. During this phase, new solutions are added to the archive, while an equal number of worse solutions are removed. This updating mechanism ensures that only the best solutions are kept in the archive, effectively guiding the search process and improving the algorithm’s exploration of the solution space [26].

## 3. The Proposed Multivariate GMM-ACO*_R_* Algorithm

The core concept of the traditional ${\mathrm{ACO}}_{R}$ algorithm lies in the use of a continuous probability density function, which is calculated based on the univariate Gaussian kernel mentioned in Section 2. However, this method samples each dimension of the solution independently, which limits its ability to take into account interdependencies or correlations between features. This limitation can lead to sub-optimal exploration of the search space, reducing the efficiency of the algorithm and potentially leading to slower convergence or deadlock in local optima, particularly for problems with high-dimensional and interacting variables. To enhance the accuracy of exploration and exploitation in the search space, this paper introduces a multivariate Gaussian Mixture Model distribution for calculating the Gaussian kernel, enabling the generation of optimal solutions.

### 3.1. Representation of Solutions

In the proposed multivariate GMM-${\mathrm{ACO}}_{R}$ algorithm, the solution space is represented using a multivariate Gaussian Mixture Model. The algorithm begins by initializing a solution archive containing a set of elite solutions, which are randomly sampled within the bounds of the variables. These solutions are evaluated using the objective function, and the archive is sorted on the basis of cost values. To more accurately model the distribution of solutions, the archive is clustered using the k-means clustering algorithm [27], where the number of clusters corresponds to the number of Gaussian components in the GMM. Subsequently, the parameters of each Gaussian component are estimated based on the data points assigned to the respective clusters. These parameters include the following:

- $\mu_{k}$: The mean vector of the *k*-th Gaussian component (cluster center).
- $\Sigma_{k}$: The covariance matrix of the *k*-th Gaussian component, capturing the relationships between variables.
- $\pi_{k}$: The weight (or mixture coefficient) of the *k*-th Gaussian component, representing the proportion of solutions in the *k*-th cluster.

After clustering, the probability density function (PDF) of a multivariate Gaussian distribution for a given solution x is computed as follows:

(4) $$N\left(x|\mu_{k},\Sigma_{k}\right)=\frac{1}{\sqrt{{\left(2\pi \right)}^{n}\det\left(\Sigma_{k}\right)}}\exp\left(-\frac{1}{2}{\left(x-\mu_{k}\right)}^{⊤}\Sigma_{k}^{-1}\left(x-\mu_{k}\right)\right)$$

where

- *x* is the solution vector.
- *n* is the dimensionality of the solution space (i.e., the number of variables).
- $\det(\Sigma_{k})$ is the determinant of the covariance matrix.
- $\Sigma_{k}^{-1}$ is the inverse of the covariance matrix.

The global probability density function (PDF) of the GMM is obtained as a weighted sum of the PDFs and its individual Gaussian components:

(5) $$P\left(x\right)=\sum_{k=1}^{K}\pi_{k}\;N\left(x|\mu_{k},\Sigma_{k}\right)$$

Hence, K is the total number of Gaussian components (clusters), and $\pi_{k}$ satisfies the constraint

(6) $$\sum_{k=1}^{K}\pi_{k}=1$$

This representation of solutions enables the algorithm to effectively balance exploration and exploitation by capturing both the global structure of the search space and the local dependencies between variables. The number of components ensures the model’s flexibility and adaptability to various problem complexities.

### 3.2. Ant-Based Solution Construction

The ant-based solution-building process constructs candidate solutions by sampling from a Gaussian Mixture Model (GMM). During each iteration, a Gaussian component is probabilistically selected using a roulette wheel mechanism, where the likelihood of choosing a component is proportional to its weight in the GMM. Once a component is selected, a candidate solution is generated by sampling from its corresponding multivariate Gaussian distribution, defined by a mean vector and a covariance matrix.

If any dimensions of the sampled solution exceed predefined variable bounds, they are clamped to the nearest valid boundary value. Each candidate solution is then evaluated via the objective function, and its resulting cost (fitness) is recorded for subsequent optimization steps.

### 3.3. Update Mechanism

Having generated new candidate solutions, the algorithm merges the current solution archive with the newly generated solutions. The combined population is then sorted according to its cost values, and the best solutions are selected to update the solution archive. GMM parameters are dynamically adjusted by re-ranking the updated archive using k-means. The means, covariance matrices, and weights of the Gaussian components are recalculated according to the new groups. This dynamic updating process allows the GMM to adapt to changes in the solution space, improving the algorithm’s ability to balance exploration and exploitation. The process is repeated for a predefined number of iterations, with the best solution being identified and saved at each iteration.The pseudo-code for the proposed multivariate GMM-${\mathrm{ACO}}_{R}$ algorithm is provided in Algorithm 1.
**Algorithm 1** MGMM-${\mathrm{ACO}}_{R}$ Algorithm Pseudo-Code  1:Initialize the population of solutions.  2:Evaluate the cost of each solution and create the archive.  3:Cluster solutions using k-means into *K* clusters.  4:Calculate the GMM parameters based on the clusters.  5:**while** i<MaxIteration **do**  6:      Each ant selects a Gaussian component using Roulette Wheel Selection.  7:      Solutions are sampled from the chosen Gaussian component using the multivariate distribution.  8:      Combine the current archive with the newly generated solutions.  9:      Sort the combined population and keep only the best nPop solutions.10:      Recompute the GMM parameters based on the updated archive.11:**end while**12:**return** Best solution.

### 3.4. Performance Evaluation

To evaluate the performance of the proposed algorithm, a set of reference functions was used [28]. These functions are classified into two categories: unimodal and multimodal. While all benchmark functions have a global minimum, multimodal functions also include multiple local optima, which makes them particularly suitable for evaluating the efficiency of metaheuristic algorithms. The search spaces for these functions were selected based on their widespread adoption in the literature, as these standard ranges facilitate fair comparisons and ensure computational feasibility. In this study, thirteen benchmark functions were used to test the performance of the proposed algorithm. A comparative analysis was then conducted to compare the proposed approach with the classical ${\mathrm{ACO}}_{R}$ algorithm and the standard ACO algorithm. The benchmark functions used are shown in Table 1.

## 4. An NMPC Controller Implemented for a 4WD/4WS Robot

### 4.1. Formulation of Trajectory Tracking Using an MPC Controller

**Figure 2:**
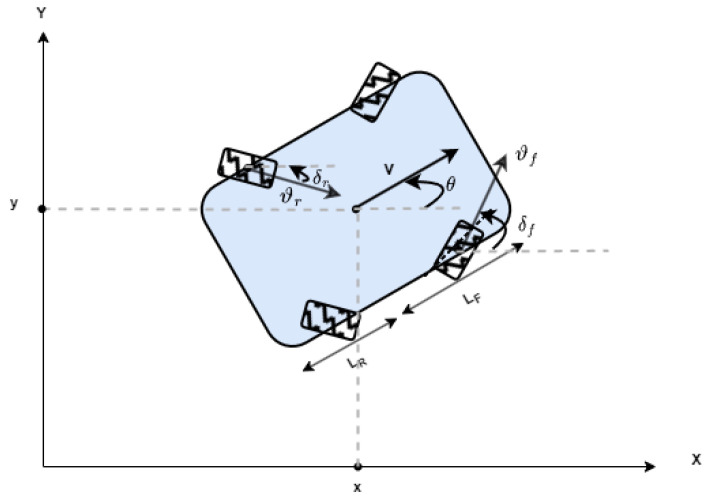
Robot’s kinematic model.

A nonlinear model predictive control (NMPC) approach was used to regulate the motion of a four-wheel steer and four-wheel drive (4WS/4WD) robot along various trajectories. The design of the NMPC controller is based on the model described in Figure 2.

The kinematic model [29] employed is defined as follows:

(7) $$\dot{x}=v\cos\left(\theta \right),\;\dot{y}=v\sin\left(\theta \right),\;\dot{\theta }=\omega ,$$

(8) $$v=\frac{v_{f}\cos\delta_{f}+v_{r}\cos\delta_{r}}{2},$$

(9) $$\omega =\frac{v(\tan\delta_{f}-\tan\delta_{r})}{L_{r}+L_{f}}.$$

with

(10) $$v_{f}=v_{r}\;and\;\delta_{f}=-\delta_{r}$$

where x and y denote the position’s robot, θ represents its heading angle, and v and ω correspond to the linear and angular velocities, respectively. Additionally, $\delta_{f}$, $v_{f}$, and $\delta_{r}$, $v_{r}$ indicate the steering angle and velocity of the front and rear wheels, respectively. Notably, $\delta_{f}$ and $v_{f}$ are defined relative to the robot’s local reference frame, while $L_{f}$ and $L_{r}$ represent the distances from the robot’s center of gravity to the front and rear axles.

At each instant, the MPC controller seeks to solve the following optimal control problem (OCP) over a receding horizon N:

(11a) $$\begin{array}{cc} \; & \underset{u\in \mathrm{admissible}}{\mathrm{minimize}}\;J_{N}\left(x_{0},u\right)=\sum_{k=0}^{N-1}\left({∥x_{u}\left(k\right)-x_{u}^{\mathrm{ref}}∥}_{Q}^{2}+{∥u\left(k\right)-u^{\mathrm{ref}}∥}_{R}^{2}\right),\\ \; & \mathrm{subject}\;\mathrm{to}: \end{array}$$

(11b) $$\begin{array}{cc} \; & \;x_{u}\left(k+1\right)=f\left(x_{u}\left(k\right),u\left(k\right)\right), \end{array}$$

(11c) $$\begin{array}{cc} \; & \;x_{u}\left(0\right)=x_{0}, \end{array}$$

(11d) $$\begin{array}{cc} \; & \;x_{u}\left(k\right)\in X,\;\forall k\in \left[0,N\right], \end{array}$$

(11e) $$\begin{array}{cc} \; & \;u(k)\in U,\;\forall k\in [0,N-1]. \end{array}$$

Hence, *x*=[x, y, θ] represents the robot’s state vector, $x^{\mathrm{ref}}$ the reference trajectory, *u* = [$v_{f}$, $\delta_{f}$] the control vector, and $u^{\mathrm{ref}}$ the desired control vector. Q and R are the weighting matrices, where Q represents the weight applied to the reference tracking states, and R penalizes the control inputs. These matrices are often set using a trial-and-error approach [30,31,32]. The main challenge lies in finding an optimal trade-off between the state of the system and the control inputs. To address this, we propose using the MGMM-${\mathrm{ACO}}_{R}$ algorithm to tune these matrices, which will be described in Section 5.

The optimal control problem is subject to a set of constraints. As described in (11b), the state dynamics constraint ensures that the optimization process remains consistent with the system’s underlying physical or mathematical model over time. The initial input constraint, detailed in (11c), ensures that the optimization problem starts from a well-defined initial state. In addition, the state constraint in (11d) and the input constraint in (11e) impose bounds on the system state and control inputs, respectively.

The OCP is solved at each time step, with the robot’s initial state being updated at each iteration based on the measured state at time step k. To solve the optimization problem, we employ a direct single-shooting method, where the discretized control inputs are used as decision variables.

### 4.2. Trajectory Tracking with Obstacle Avoidance

Obstacle avoidance is a critical component of robotic navigation, especially when a robot is required to follow a predefined trajectory. In dynamic environments, where obstacles may appear and obstruct the robot’s path [33], it becomes essential for the robot to detect and avoid these obstacles while still adhering to its desired trajectory. For simplicity, an obstacle can be modeled as a circular region encompassing the robot’s dimensions. To ensure both collision-free navigation and accurate path tracking, a model predictive control (MPC) framework is employed. In this context, the optimal control problem (OCP) is defined as follows:

(12a) $$\begin{array}{cc} \; & \underset{u\in \mathrm{admissible}}{\mathrm{minimize}}\;J_{N}\left(x_{0},u\right)=\sum_{k=0}^{N-1}\left({∥x_{u}\left(k\right)-x_{u}^{\mathrm{ref}}∥}_{Q}^{2}+{∥u\left(k\right)-u^{\mathrm{ref}}∥}_{R}^{2}\right),\\ \; & \mathrm{subject}\;\mathrm{to} \end{array}$$

(12b) $$\begin{array}{cc} \; & \;x_{u}\left(k+1\right)=f\left(x_{u}\left(k\right),u\left(k\right)\right), \end{array}$$

(12c) $$\begin{array}{cc} \; & \;x_{u}\left(0\right)=x_{0}, \end{array}$$

(12d) $$\begin{array}{cc} \; & \;x_{u}\left(k\right)\in X,\;\forall k\in \left[0,N\right], \end{array}$$

(12e) $$\begin{array}{cc} \; & \;u(k)\in U,\;\forall k\in [0,N-1]. \end{array}$$

(12f) $$\begin{array}{cc} \; & \;\sqrt{{\left(x_{\mathrm{rob}}-x_{\mathrm{obs}}\right)}^{2}+{\left(y_{\mathrm{rob}}-y_{\mathrm{obs}}\right)}^{2}}\geq r_{\mathrm{rob}}+r_{\mathrm{obs}}+d_{\min} \end{array}$$

The OCP is subject to previously defined constraints, with an additional constraint of obstacle avoidance (12f). This constraint requires that the Euclidean distance between the robot’s position and the obstacle is greater than the sum of the radius of the circular regions surrounding both the robot and the obstacle. To provide additional safety, a predefined safety distance $d_{\min}$, is incorporated into this condition, ensuring reliable collision avoidance. To solve the optimization problem, we employ a multiple-shooting method.

### 4.3. Casadi Framework

The challenges posed by the problems of optimal control of trajectory tracking and obstacle avoidance with trajectory tracking arise from their intrinsically nonlinear nature and the presence of complex constraints. Solving these problems requires a robust computational framework capable of handling these complexities efficiently. To this end, we used the CasADi framework (version 3.6.3 for MATLAB 2021b), a state-of-the-art open-source tool widely recognized for its versatility in numerical optimization and algorithmic differentiation [34]. CasADi excels at formulating and solving constrained nonlinear optimization problems, making it well suited to sophisticated control tasks in dynamic and uncertain environments.

## 5. Adaptative NMPC Controller Using MGMM-ACO*_R_* Algorithm

The most critical aspect of MPC controller design lies in the correct setting of the Q and R weighting matrices. The Q matrix determines the weight applied to an error in the reference tracking of system states, while R penalizes the magnitude or rate of change of control inputs. These weighting matrices play a crucial role in determining overall controller performance, as they directly influence the trade-off between tracking accuracy and control effort.

Setting these parameters is a complex task that often demands significant time and effort to achieve the optimal balance. To determine the appropriate trade-off that allows the system to achieve accurate reference tracking performance while ensuring stability, robustness, and efficient control effort, the MGMM-${\mathrm{ACO}}_{R}$ algorithm was employed to tune both weighting matrices.

The objective function to be optimized, by the MGMM-${\mathrm{ACO}}_{R}$ algorithm, was defined as the combination of three key elements: the integral of the time-weighted absolute error (ITAE), the MPC controller’s control effort, and the MPC controller’s average computation time.

The ITAE, as defined in Equation (13), measures the time-weighted absolute error between the system’s output and the reference trajectory. For the mobile robot, the ITAE is calculated for the x, y, and θ components, representing its position and orientation. Incorporating this metric into the optimization process ensures that tracking errors over time are minimized across all these components, enhancing the robot’s accuracy in following the desired trajectory.

(13) $$ITAE=\int_{0}^{T}t\left|e(t)\right|dt$$

In the MPC controller objective function (Equation (12a)), control effort (u) is used to generate optimized control inputs, ensuring efficient energy use while maintaining desired system performance. The dimension of the control effort vector is determined by the number of control input variables and the prediction horizon N. This term is incorporated into the objective function to minimize energy consumption across the considered scenarios.

The average computation time of the MPC controller, $(t_{\mathrm{MPC}\_\mathrm{average}}$), is included to account for computational efficiency. Since MPC requires solving optimization problems in real time, reducing computation time is essential to ensure the controller operates effectively within the system’s time constraints.

Consequently, the objective function of the optimization problem is formulated as follows:

(14) $$f=\left(ITAE_{x}+ITAE_{y}+ITAE_{\theta }\right)+w_{1}u+w_{2}t_{\mathrm{MPC}\_\mathrm{average}}$$

Prior to the optimization process, each term in the objective function is normalized to ensure proportional contribution from all components. The resulting cost function ff is then formulated and prepared for optimization using the proposed algorithm. This cost function consists of three distinct elements, each assigned a specific weighting factor. These weights enable the prioritization of particular performance criteria based on the requirements of the control task. By adjusting the weighting factors, the influence of each component can be tailored to suit the characteristics and objectives of the scenario under analysis.

## 6. Simulation Results and Comparison

### 6.1. Benchmarking Evaluation

This subsection provides a comparative evaluation of the proposed MGMM-${\mathrm{ACO}}_{R}$ algorithm, highlighting its performance through comparative analysis. The evaluation is carried out using various benchmark functions, including unimodal and multimodal functions, to assess the algorithm’s adaptability and robustness. For comparative analysis, we compare the results obtained by the MGMM-${\mathrm{ACO}}_{R}$ algorithm with those obtained by the ACOstandard algorithm and the ${\mathrm{ACO}}_{R}$ conventional algorithm. Table 2 presents a detailed comparison of the performance of each algorithm for the selected reference functions.

The tests are conducted on a computer with the following specifications: Windows 10 (64-bit) as the operating system, MATLAB R2021b as the software environment, and hardware comprising an Intel(R) Core(TM) i7-10750H CPU @ 2.60 GHz (2.59 GHz) and 16.0 GB of RAM.

The three algorithms were compared under identical conditions to ensure a fair and consistent evaluation. The dimension of the solution space was set to n = 30, and the number of decision variables was set to k = 36. For the ${\mathrm{ACO}}_{R}$ algorithm, the selection pressure parameter was varied in the interval [0.0001, 0.5]. The number of clusters (K) was determined using a trial-and-error approach. For the functions evaluated, the K values tested were (K=3,6,10,20), with the optimum value selected on the basis of performance.

As indicated in Table 2, the proposed method outperforms the other two algorithms for all reference functions. The MGMM-${\mathrm{ACO}}_{R}$ algorithm consistently outperformed its counterparts for both unimodal and multimodal functions. In particular, for multimodal functions, its optimization capabilities are clearly superior to those of the ${\mathrm{ACO}}_{R}$ algorithm.

This performance improvement can be attributed to the limitations of the ${\mathrm{ACO}}_{R}$ algorithm, which struggles to optimize multimodal functions due to its approach of treating variables independently during the optimization process. In contrast, the proposed method introduces a mechanism for capturing and exploiting relationships between variables, enabling more efficient optimization, particularly for complex multimodal problems.

**Figure 3:**
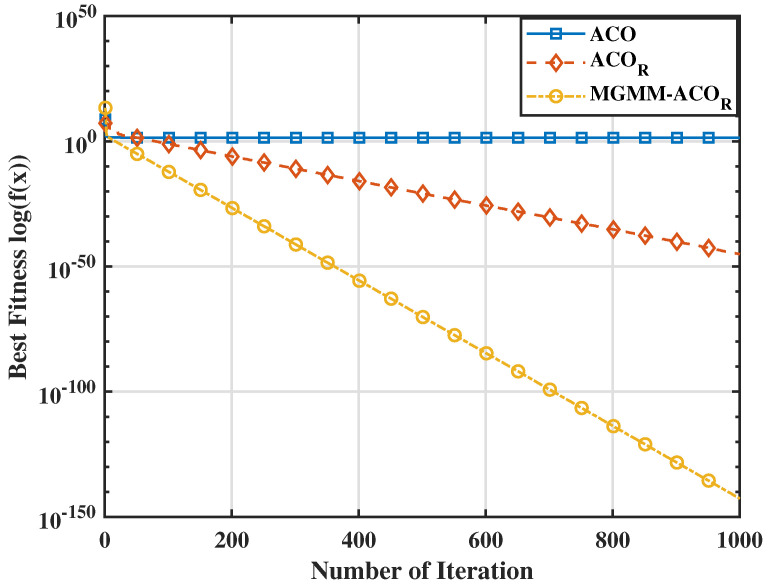
Fitnessvalue for the Schwefel 2.22 function.

**Figure 4:**
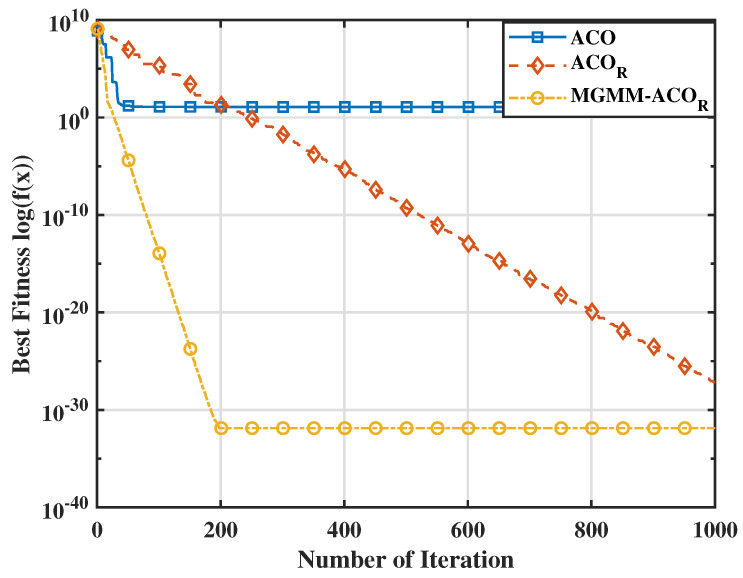
Fitnessvalue for the Penalized 2 function.

**Figure 5:**
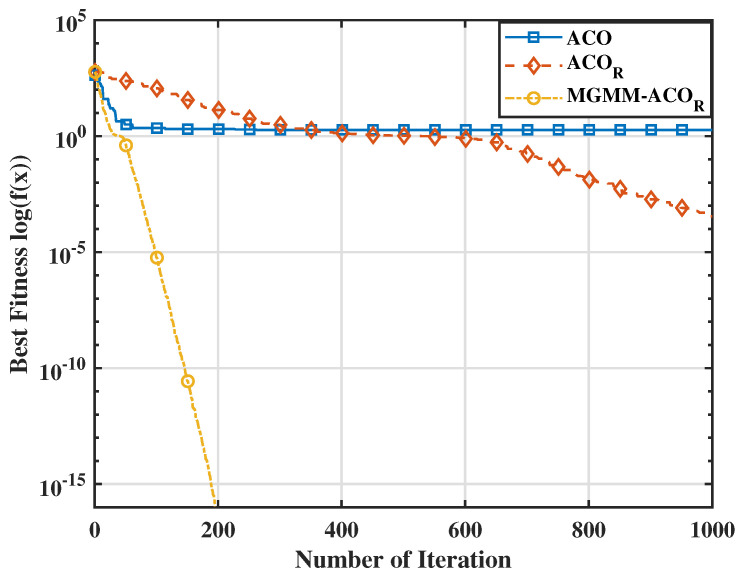
Fitness value for the Griewank function.

**Figure 6:**
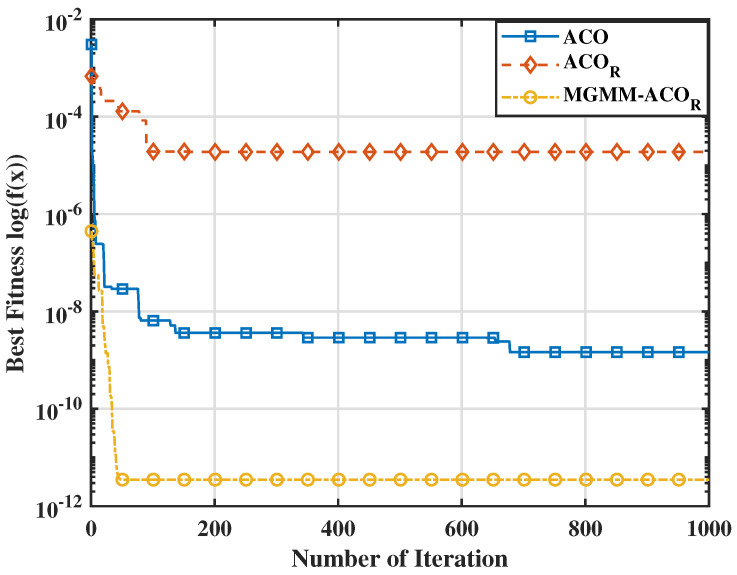
Fitness value for the Xin-She Yang function.

To further substantiate the effectiveness of the proposed MGMM-${\mathrm{ACO}}_{R}$ algorithm, we carried out a comparative study with particle swarm optimization (PSO) and its advanced variants. Notably, when evaluated against the most recent PSO variant, HT-PSO [35], the MGMM-${\mathrm{ACO}}_{R}$ algorithm consistently demonstrated superior performance over a wide range of benchmark functions. The results indicate that the proposed method converges rapidly while perfectly avoiding local optima, underlining its effectiveness in solving complex optimization problems. The aforementioned interpretations are further supported by the variation in average optimum fitness values (on a logarithmic scale) illustrated in Figure 3, Figure 4, Figure 5 and Figure 6. These figures demonstrate the superior convergence behavior of the proposed MGMM-${\mathrm{ACO}}_{R}$ algorithm compared to other algorithms.

The logarithmic representation of the fitness values highlights the significant performance gap, particularly for multimodal functions, for which the ${\mathrm{ACO}}_{R}$ algorithm shows slower convergence and struggles to escape local optima due to its inability to take variable interdependencies into account.

By contrast, the proposed algorithm leverages its capability to model and exploit relationships between variables, enabling a more robust exploration and exploitation of the solution space.

### 6.2. Trajectory Tracking and Dynamic Obstacle Avoidance

The newly proposed MGMM-${\mathrm{ACO}}_{R}$ algorithm was applied to a 4WD/4WS mobile robot and compared with the conventional ${\mathrm{ACO}}_{R}$ algorithm. Both algorithms were evaluated in multiple scenarios, including circular and eight-trajectory tracking and dynamic obstacle avoidance. All scenarios were tested using a fixed sampling time of $T_{s}$ = 0.1 s. The parameters of both algorithms, as well as the optimized values of Q and R, are presented in Table 3.

**Figure 7:**
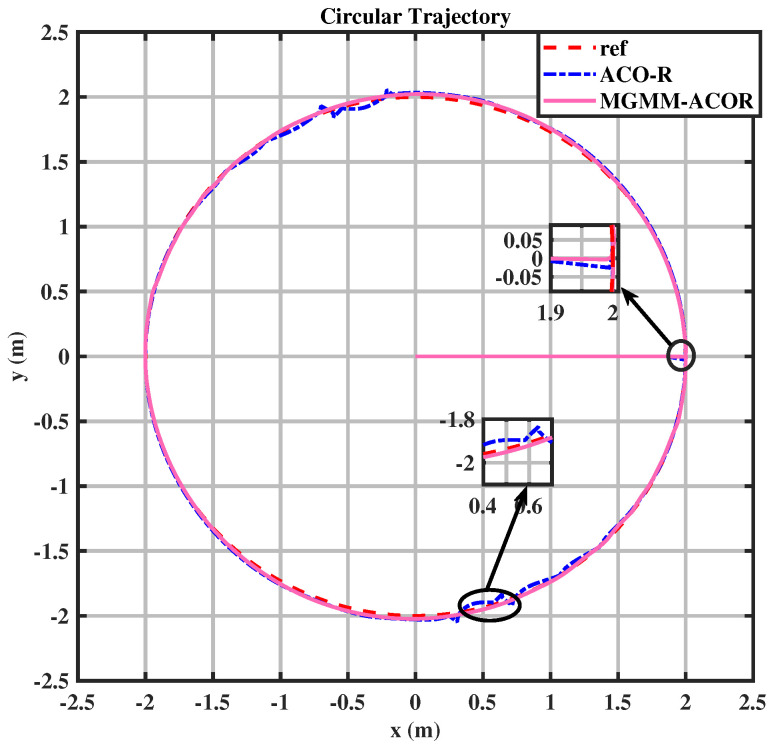
Circular trajectory.

**Figure 8:**
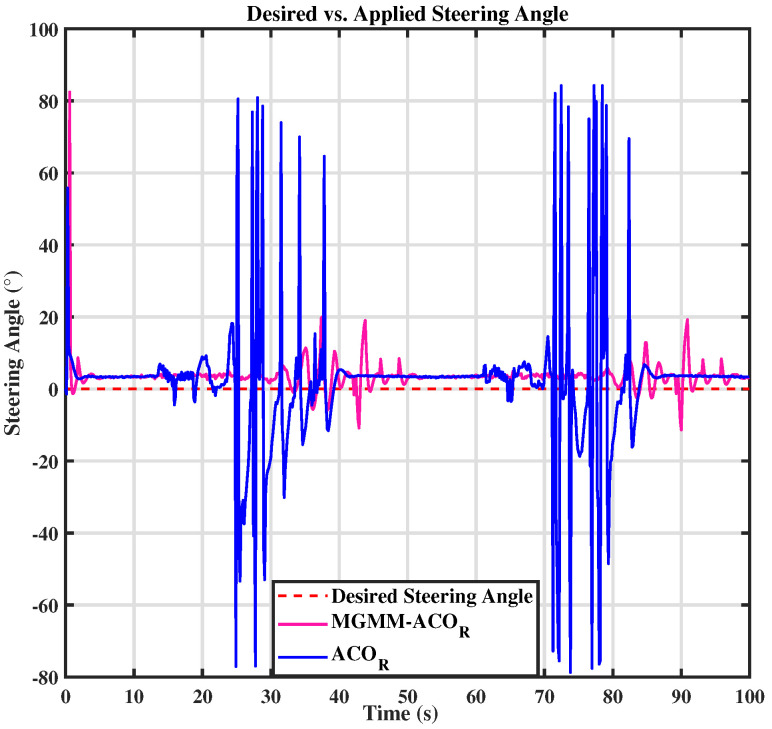
Desired vs. calculated steering angle.

**Figure 9:**
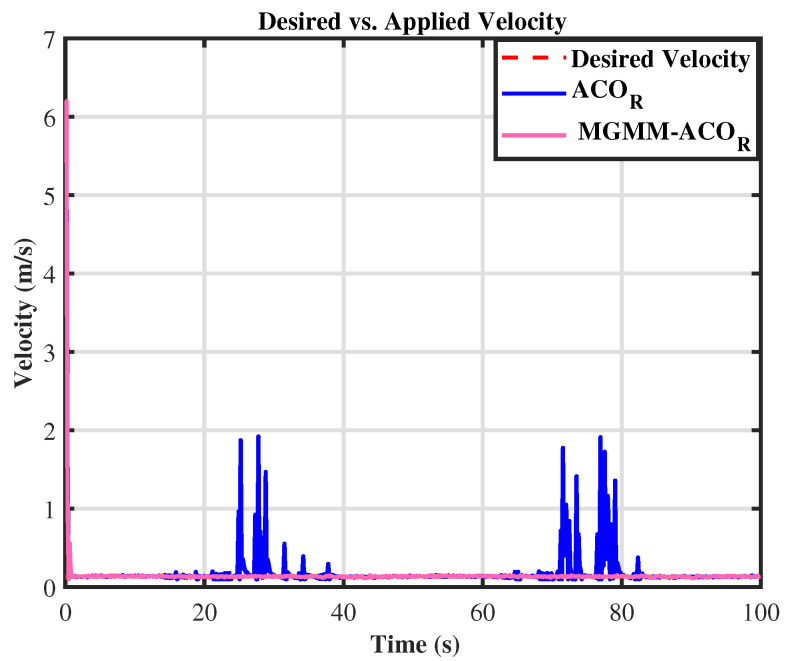
Desired vs. calculated velocity.

For the circular trajectory, illustrated in Figure 7, the MPC controller parameters were set as follows: a prediction horizon time of T = 0.15 s and a prediction step count of N = 10. The weighting matrices were optimized using both of the previously mentioned algorithms. The MGMM-${\mathrm{ACO}}_{R}$ algorithm demonstrated significantly superior tracking performance compared to ${\mathrm{ACO}}_{R}$. It was the first to converge to the trajectory and maintained stable and precise tracking throughout. The results are detailed in Figure 8 and Figure 9, where the performance and control actions during the circular path are illustrated.

**Figure 10:**
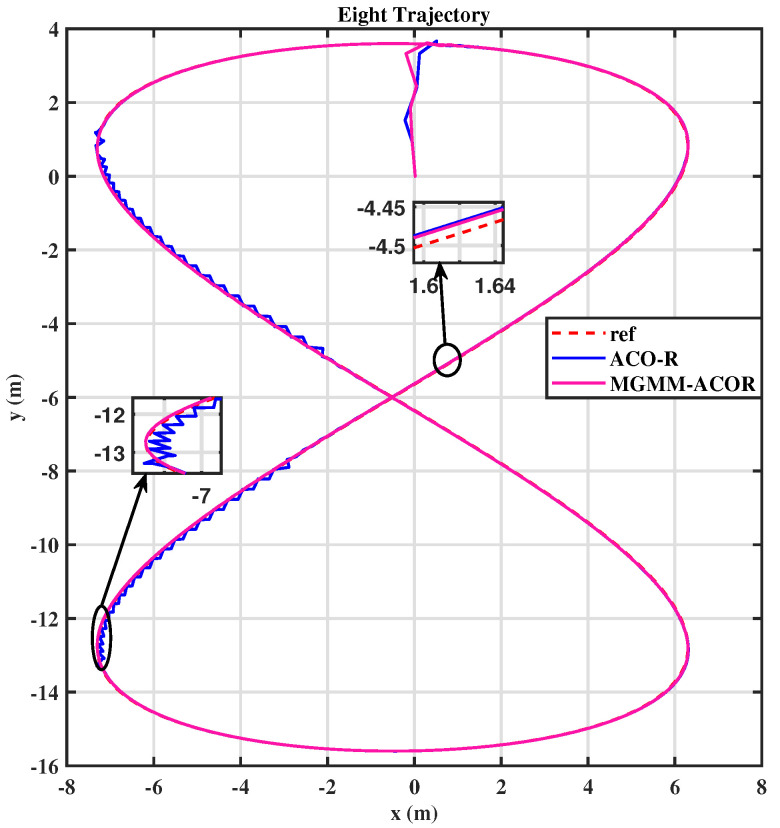
Eight trajectory.

**Figure 11:**
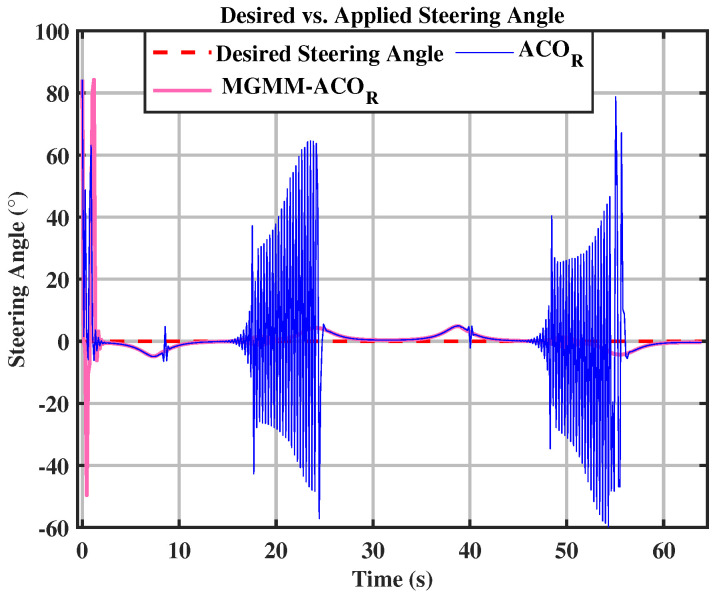
Desired vs. calculated steering angle.

**Figure 12:**
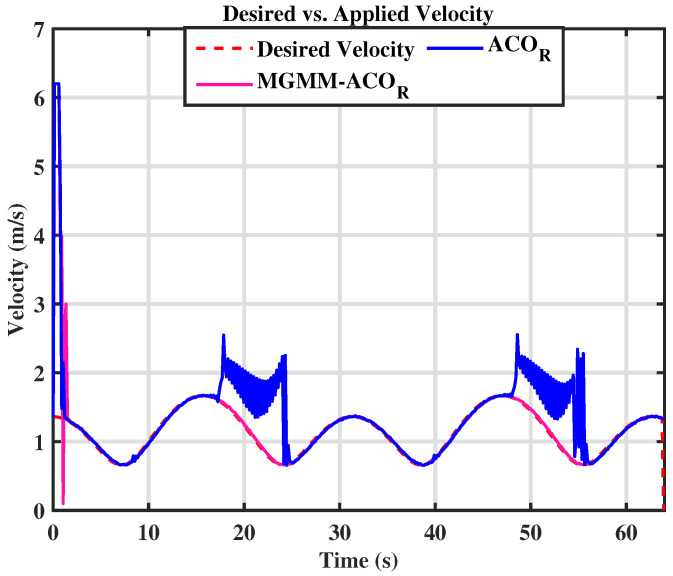
Desired vs. calculated velocity.

For the eight trajectory shown in Figure 10, the MPC controller parameters were set as follows: a prediction horizon time of T = 0.15 s and a prediction step count of N = 15. The MPC controller’s weighting matrices, optimized by the MGMM-${\mathrm{ACO}}_{R}$ algorithm, ensured superior accuracy and stability. Initially, the algorithm worked to align the robot with the reference trajectory and progressively improved tracking precision. It effectively handled the complex curvature of turning maneuvers, maintaining consistent trajectory adherence. The performance during this scenario is illustrated in Figure 11 and Figure 12.

In both the circular and eight trajectories, the ${\mathrm{ACO}}_{R}$ algorithm exhibited instability, particularly during turns, where significant fluctuations caused deviations from the reference trajectory. These oscillations disrupted the robot’s ability to maintain the required velocity and steering angles, leading to inconsistencies in motion control.

**Figure 13:**
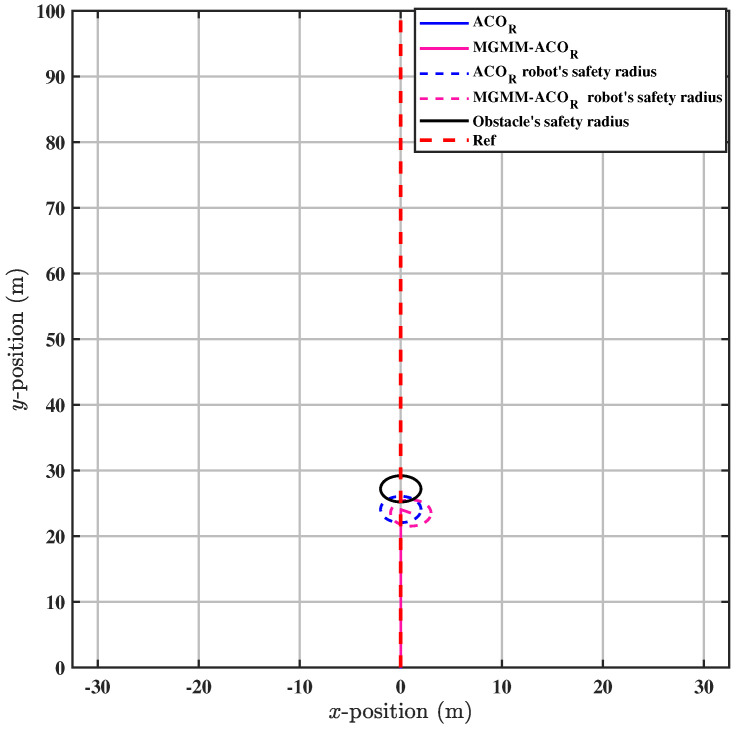
Avoiding the dynamic obstacle.

To further validate the accuracy of the proposed method, we conducted tests in a scenario involving a dynamic obstacle, where the robot was required to avoid the obstacle while maintaining adherence to the trajectory, as illustrated in Figure 13. The MPC controller parameters were configured with a prediction horizon time of T = 0.18 s and a prediction step count of N = 18.

The results indicate that the weighting matrices of the MPC controller, optimized by MGMM-${\mathrm{ACO}}_{R}$, enabled the robot to successfully avoid the obstacle first. On the other hand, the ${\mathrm{ACO}}_{R}$ algorithm initially entered the safe zone, and then avoided the obstacle.

**Figure 14:**
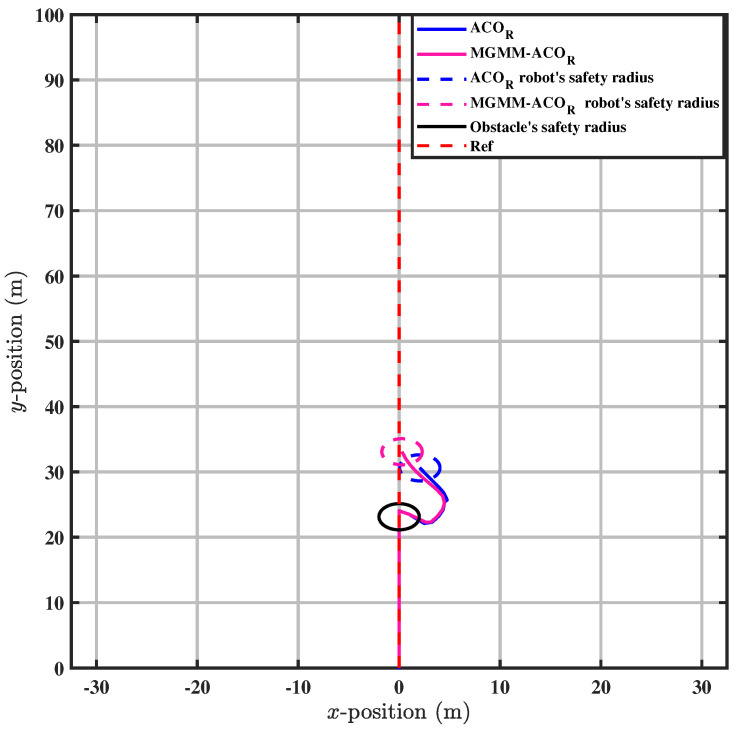
Enteringthe path after avoiding the obstacle.

Once the obstacle had been avoided, the proposed method proved to be superior again by being the first to maintain the trajectory tracking, as illustrated in Figure 14.

**Figure 15:**
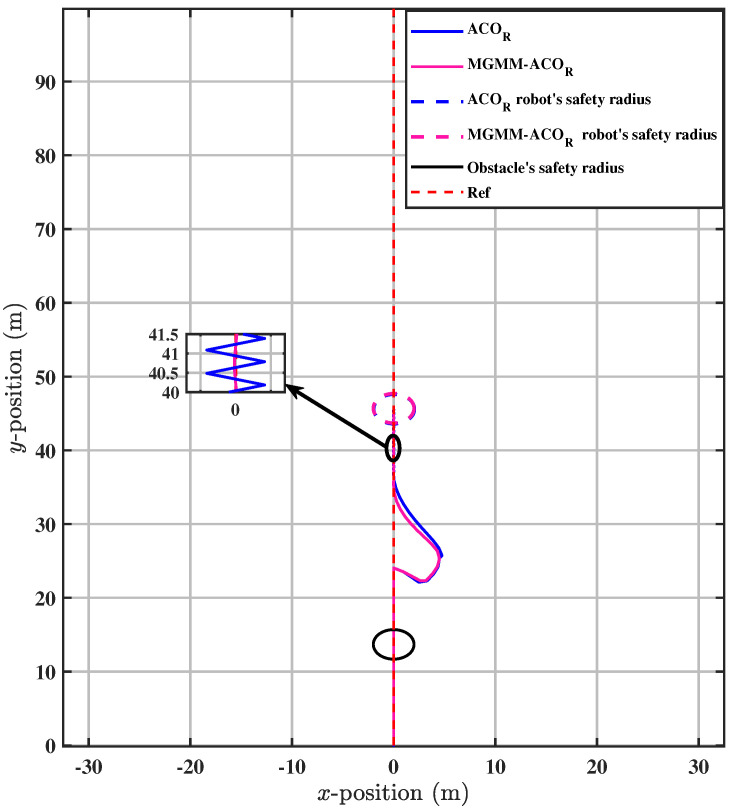
Maintaining path tracking.

**Figure 16:**
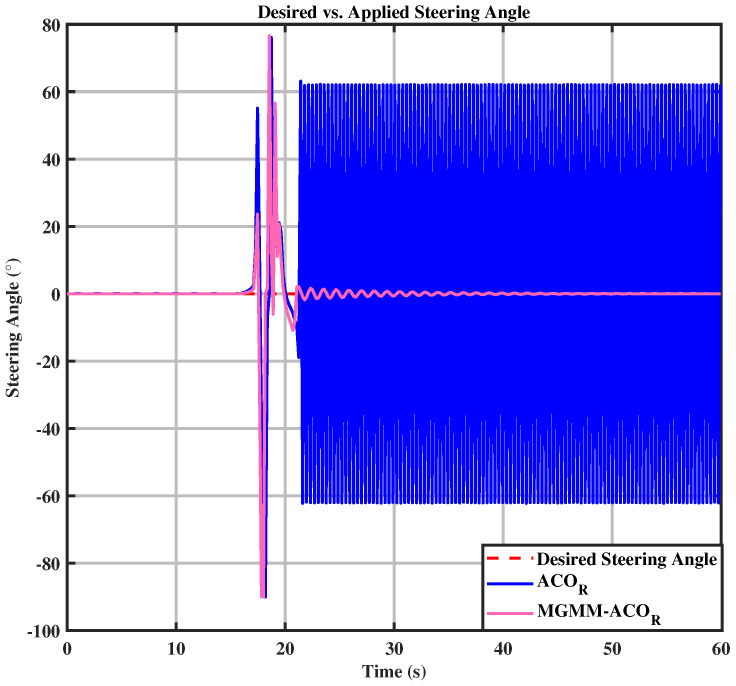
Desired vs. calculated steering angle.

**Figure 17:**
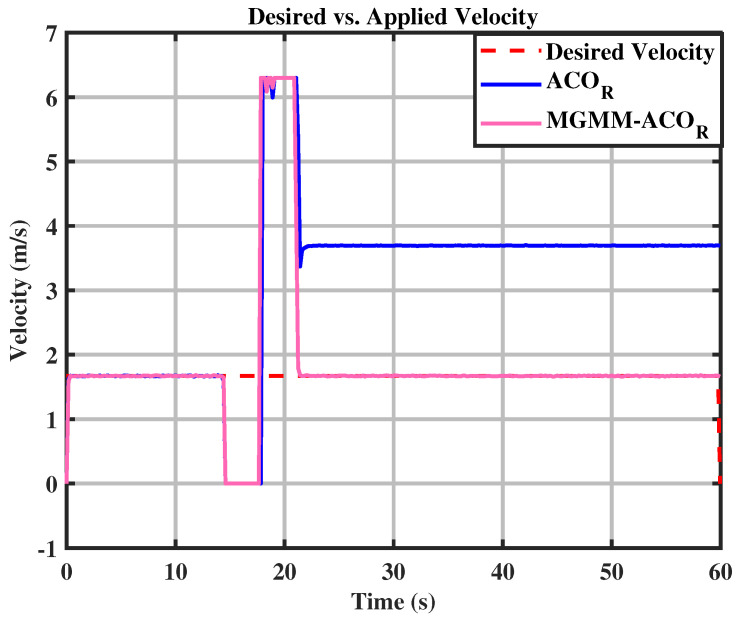
Desiredvs. calculated velocity.

Upon zooming in on the trajectory after both algorithms have maintained path tracking, it becomes evident that the ${\mathrm{ACO}}_{R}$ algorithm struggles to follow the trajectory. As depicted in Figure 15, significant fluctuations around the trajectory are observed. This can be further substantiated by Figure 16 and Figure 17, where it is clear that the novel algorithm successfully tracks the desired velocity, while a notable gap exists between the desired and the actual velocity calculated by the classical ${\mathrm{ACO}}_{R}$ algorithm.

As presented in Table 4, the parameters composing the objective function were evaluated separately after independently injecting the optimized variables (derived from both algorithms) into the MPC controller. This comparative analysis highlights the effectiveness of our proposed method, which optimizes the system variables while explicitly considering their interdependencies.

For the circular trajectory-tracking scenario, the conventional MPC-${\mathrm{ACO}}_{R}$ obtained ${\mathrm{ITAE}}_{x}$ and ${\mathrm{ITAE}}_{\theta }$ values of 0.31329 and 0.36536, respectively. However, these performances were achieved at the cost of significantly increased control effort and prolonged computation time in the MPC framework. These inefficiencies probably contributed to the observed fluctuations in trajectory tracking, in Figure 7, as the controller prioritized minimizing tracking errors at the expense of stability and computational efficiency. On the contrary, the proposed MPC-MGMM-${\mathrm{ACO}}_{R}$ method achieves an optimal balance between all parameters, including control effort and computation time-critical factors for real-world controller performance. By explicitly accounting for these trade-offs, our approach enables smoother trajectory tracking while maintaining computational trackability.

Similarly, for the eight trajectory, the classical algorithm works by optimizing variables in isolation, neglecting their inherent correlations. This neglect directly compromises the behavioral consistency of the MPC controller. In contrast, the proposed method systematically addresses this limitation by explicitly modeling the interdependencies of the variables, thereby ensuring a coordinated optimization that aligns with the controller’s operational constraints.

In the scenario of dynamic obstacle avoidance with trajectory tracking, a multimodal optimization problem, consideration of the correlations between the optimized variables is essential. Neglecting these interdependencies can lead to collisions with obstacles or violations of their safety boundaries, as shown in Figure 13 for the conventional ${\mathrm{ACO}}_{R}$ method. The conventional approach does not systematically coordinate variables, leading to dangerous trajectories. In contrast, the proposed MGMM-${\mathrm{ACO}}_{R}$ framework remedies this limitation by explicitly integrating variable correlations into its optimization process. This ensures robust trajectory tracking while simultaneously minimizing all objective function measures, including collision avoidance critical to safety and computational efficiency, as validated in Table 4.

## 7. Conclusions

In this study, we have proposed an improved optimization approach, MGMM-${\mathrm{ACO}}_{R}$, designed to improve the performance of nonlinear model predictive control (MPC) for trajectory tracking in a 4WD/4WS mobile robot. The proposed algorithm addresses key limitations of the classical ${\mathrm{ACO}}_{R}$ by explicitly modeling variable interdependencies using a multivariate Gaussian Mixture Model whose parameters are efficiently estimated via k-means clustering. This integration significantly improves the balance between exploration and exploitation during the optimization process.

The effectiveness of MGMM-${\mathrm{ACO}}_{R}$ was validated by a two-phase evaluation. First, benchmark function experiments demonstrated superior convergence speed and solution quality compared to well-known algorithms, including PSO, its variants, ACO, and ${\mathrm{ACO}}_{R}$. Second, the algorithm was integrated into an MPC framework and tested in various scenarios, such as circular and eight-trajectory tracking, as well as dynamic obstacle avoidance. The results revealed significant improvements in tracking accuracy, control effort, and computational efficiency, confirming the robustness and practical applicability of the proposed method in dynamic environments.

As future work, we intend to extend the framework by incorporating the dynamic model of the robot to more accurately reflect real-world conditions. Additionally, we aim to investigate the system’s performance in more complex environments involving multiple dynamic obstacles.

## Figures and Tables

**Table 1:** Benchmark functions: unimodal and multimodal.

Unimodal Functions		
Function Name	Mathematical Expression	Search Space
Sphere	$f_{1}\left(x\right)=\sum_{i=1}^{d}{\left(x_{i}\right)}^{2}$	[−100,100]
Schwefel 2.21	$f_{2}\left(x\right)=\max_{i}\left\{\right|x_{i}\left|,1\leq i\leq d\right\}$	[−100,100]
Schwefel 2.22	$f_{3}\left(x\right)=\sum_{i=1}^{d}|x_{i}|+\prod_{i=1}^{d}\left|x_{i}\right|$	[−10,10]
Schwefel 1.2	$f_{4}\left(x\right)=\sum_{i=1}^{d}{\left(\sum_{j=1}^{i}x_{j}\right)}^{2}$	[−100,100]
Quartic Noise	$f_{5}\left(x\right)=\sum_{i=1}^{d}ix_{i}^{4}+\mathrm{rand}\left(0,1\right)$	[−1.28,1.28]
Rosenbrock	$f_{6}\left(x\right)=\sum_{i=1}^{d-1}\left[100{\left(x_{i+1}-x_{i}^{2}\right)}^{2}+{\left(x_{i}-1\right)}^{2}\right]$	[−30,30]
Multimodal Functions		
Penalized 2	$f_{7}\left(x\right)=\frac{1}{10}\left[\sin^{2}\left(3\pi x_{1}\right)+\sum_{i=1}^{d-1}{\left(x_{i}-1\right)}^{2}\left(1+\sin^{2}\left(3\pi x_{i+1}\right)\right)+{\left(x_{d}-1\right)}^{2}\left(1+\sin^{2}\left(2\pi x_{d}\right)\right)+\sum_{i=1}^{d}u\left(x_{i},5,100,4\right)\right]$	[−50,50]
Penalized 1	$f_{8}\left(x\right)=\frac{\pi }{d}\left[10\sin^{2}\left(\pi y_{1}\right)+\sum_{i=1}^{d-1}{\left(y_{i}-1\right)}^{2}\left(1+10\sin^{2}\left(\pi y_{i+1}\right)\right)+{\left(y_{d}-1\right)}^{2}\right]+\sum_{i=1}^{d}u\left(x_{i},10,100,4\right),\;y_{i}=1+\frac{x_{i}+1}{4}$	[−50,50]
Griewank	$f_{9}\left(x\right)=\sum_{i=1}^{d}\frac{x_{i}^{2}}{4000}-\prod_{i=1}^{d}\cos\left(\frac{x_{i}}{\sqrt{i}}\right)+1$	[−600,600]
Rastrigin	$f_{10}\left(x\right)=10d+\sum_{i=1}^{d}\left[x_{i}^{2}-10\cos\left(2\pi x_{i}\right)\right]$	[−5.12,5.12]
Ackley	$f_{11}\left(x\right)=-ae^{-b\sqrt{\frac{1}{d}\sum_{i=1}^{d}x_{i}^{2}}}-e^{\frac{1}{d}\sum_{i=1}^{d}\cos\left(cx_{i}\right)}+a+e$	[−32,32]
Salomon	$f_{12}\left(x\right)=1-\cos\left(2\pi \sqrt{\sum_{i=1}^{d}x_{i}^{2}}\right)+0.1\sqrt{\sum_{i=1}^{d}x_{i}^{2}}$	[−20,20]
Xin-She Yang	$f_{13}\left(x\right)=\sum_{i=1}^{d}\left|x_{i}\right|e^{-\sum_{i=1}^{d}\sin^{2}\left(x_{i}\right)}$	[−5,5]

**Table 2:** Simulation results of benchmarking evaluation.

Function	ACO	${\mathrm{ACO}}_{R}$	MGMM-${\mathrm{ACO}}_{R}$
$f_{1}$	$1.9847\times 10^{2}$	$4.6853\times 10^{-31}$	$1.04\times 10^{-202}$
$f_{2}$	$4.6523\times 10^{0}$	$5.3085\times 10^{-7}$	$8.4506\times 10^{-50}$
$f_{3}$	$2.4669\times 10^{1}$	$7.6315\times 10^{-46}$	$2.1667\times 10^{-143}$
$f_{4}$	$3.0538\times 10^{2}$	$2.0801\times 10^{-15}$	$1.2455\times 10^{-55}$
$f_{5}$	$1.8687\times 10^{1}$	$9.2135\times 10^{-6}$	$1.2094\times 10^{-6}$
$f_{6}$	$5.4482\times 10^{1}$	$5.1921\times 10^{0}$	$5.1204\times 10^{-4}$
$f_{7}$	$1.2005\times 10^{1}$	$7.4703\times 10^{-28}$	$1.3498\times 10^{-32}$
$f_{8}$	$1.9590\times 10^{1}$	$2.9887\times 10^{-29}$	$4.9992\times 10^{-33}$
$f_{9}$	$1.8632\times 10^{0}$	$3.5059\times 10^{-4}$	$0.0\times 10^{0}$
$f_{10}$	$5.4051\times 10^{1}$	$1.4542\times 10^{2}$	$0.0\times 10^{0}$
$f_{11}$	$4.1450\times 10^{0}$	$7.9936\times 10^{-15}$	$4.4409\times 10^{-15}$
$f_{12}$	$4.9987\times 10^{-1}$	$1.9987\times 10^{-1}$	$9.9873\times 10^{-3}$
$f_{13}$	$1.4608\times 10^{-9}$	$1.1466\times 10^{-7}$	$3.5124\times 10^{-12}$

**Table 3:** Algorithm’s parameters and corresponding best solutions.

	Algorithm’s Parameters		Best Solution	
	MGMM-${\mathrm{ACO}}_{R}$	${\mathrm{ACO}}_{R}$	MGMM-${\mathrm{ACO}}_{R}$	${\mathrm{ACO}}_{R}$
Circular trajectory	K = 3 MaxIter = 100 nvar = 5 npop = 150 Samples = 250	q = 0.5, z = 1 MaxIter = 100 nvar = 5 npop = 150 Samples = 250	Q = diag(800, 82.6308, 0) R = diag(0.36327, 0.0992)	Q = diag(800, 97.5706, 5.0509 × 10^−7^) R = diag(0.2000, 0.2973)
Eight trajectory	K = 3 MaxIter = 60 nvar = 5 npop = 100 Samples = 200	q = 0.5, z = 1 MaxIter = 60 nvar = 5 npop = 100 Samples = 200	Q = diag(10, 199.27, 0) R = diag(0.20, 0.9775)	Q = diag(57.5204, 184.03, 1.0636 × 10^−5^) R = diag(0.20, 0.8701)
Dynamic obstacle avoidance	K = 3 MaxIter = 60 nvar = 6 npop = 150 Samples = 250	q = 0.5, z = 1 MaxIter = 60 nvar = 6 npop = 150 Samples = 250	Q = diag(196.36, 101.40, 5.2519 × 10^−5^, 2.2356 × 10^−7^) R = diag(0.1676, 0.7030)	Q = diag(153.95, 110.81, 1 × 10^−4^, 1 × 10^−6^) R = diag(0.10, 0.8642)

**Table 4:** Comparison of simulation outcomes.

		MPC-${\mathrm{ACO}}_{R}$	MPC-MGMM-${\mathrm{ACO}}_{R}$
Circular	${\mathrm{ITAE}}_{x}$ ${\mathrm{ITAE}}_{y}$ ${\mathrm{ITAE}}_{\theta }$ Control Effort $t_{MPC\_average}$	0.31329 0.26522 0.36536 0.25973 1.6561 × 10^−5^	0.35465 0.25244 0.36538 0.06775 1.3474 × 10^−5^
Eight	${\mathrm{ITAE}}_{x}$ ${\mathrm{ITAE}}_{y}$ ${\mathrm{ITAE}}_{\theta }$ Control Effort $t_{MPC\_average}$	0.18497 0.24437 0.49605 0.10342 1.6505 × 10^−5^	0.22567 0.23974 0.43881 0.095839 1.4086 × 10^−5^
Dynamic Obstacle	${\mathrm{ITAE}}_{x}$ ${\mathrm{ITAE}}_{y}$ ${\mathrm{ITAE}}_{\theta }$ Control Effort $t_{MPC\_average}$	0.036835 0.073705 0.4307 0.76467 2.0863 × 10^−5^	0.028935 0.059707 0.48267 0.15837 1.8541 × 10^−5^

## Data Availability

Data is contained within the article.

## References

[1] Paz-Delgado G.J., Sánchez-Ibáñez J.R., Domínguez R., Pérez-Del-Pulgar C.J., Kirchner F., García-Cerezo A. (2023). Combined Path and Motion Planning for Workspace Restricted Mobile Manipulators in Planetary Exploration. IEEE Access.

[2] Li M., Wu F., Wang F., Zou T., Li M., Xiao X. (2024). CNN-MLP-Based Configurable Robotic Arm for Smart Agriculture. Agriculture.

[3] Morsi N.M., Mata M., Harrison C.S., Semple D. (2024). Adaptive robotic system for the inspection of aerospace slat actuator mount. Front. Robot..

[4] Mortensen A.B., Pedersen E.T., Benedicto L.V., Burg L., Madsen M.R., Bøgh S. Two-Stage Reinforcement Learning for Planetary Rover Navigation: Reducing the Reality Gap with Offline Noisy Data. Proceedings of the International Conference on Space Robotics (iSpaRo).

[5] Cui L., Le F., Xue X., Sun T., Jiao Y. (2024). Design and experiment of an agricultural field management robot and its navigation control system. Agronomy.

[6] Le T.L., Khang N.G., Thien V.D. (2025). A Study on the Kalman Filter Based PID Controller for Mecanum-wheeled Mobile Robot. J. Phys. Conf. Ser..

[7] Thai N.H., Ly T.T.K., Thien H., Dzung L.Q. (2022). Trajectory tracking control for differential-drive mobile robot by a variable parameter PID controller. Int. J. Mech. Eng. Robot. Res..

[8] Huang H., Gao J. (2024). Backstepping and novel sliding mode trajectory tracking controller for wheeled mobile robots. Mathematics.

[9] Pérez-Juárez J.G., García-Martínez J.R., Barra-Vázquez O.A., Cruz-Miguel E.E., Olmedo-García L.F., Santiago A.M. Fuzzy logic controller for a terrestrial mobile robot based on the global positioning system. Proceedings of the IEEE International Conference on Engineering Veracruz (ICEV).

[10] Ait Dahmad H., Ayad H., Cerezo A.G., Mousannif H. (2024). IT-2 Fuzzy Control and Behavioral Approach Navigation System for Holonomic 4WD/4WS Agricultural Robot. Int. J. Comput. Commun. Control..

[11] Kamil F., Moghrabiah M.Y. (2022). Multilayer decision-based fuzzy logic model to navigate mobile robot in unknown dynamic environments. Fuzzy Inf. Eng..

[12] Shivkumar S., Amudha J., Nippun Kumaar A.A. (2024). Federated deep reinforcement learning for mobile robot navigation. J. Intell. Fuzzy Syst..

[13] Xiao H., Chen C., Zhang G., Chen C.P. (2024). Reinforcement learning-driven dynamic obstacle avoidance for mobile robot trajectory tracking. Knowl.-Based Syst..

[14] Cheng X., Zhang S., Cheng S., Xia Q., Zhang J. (2022). Path-following and obstacle avoidance control of nonholonomic wheeled mobile robot based on deep reinforcement learning. Appl. Sci..

[15] Schwenzer M., Ay M., Bergs T., Abel D. (2021). Review on model predictive control: An engineering perspective. Int. J. Adv. Manuf. Technol..

[16] Zhang K., Wang J., Xin X., Li X., Sun C., Huang J., Kong W. (2022). A survey on learning-based model predictive control: Toward path tracking control of mobile platforms. Appl. Sci..

[17] Katona K., Neamah H.A., Korondi P. (2024). Obstacle avoidance and path planning methods for autonomous navigation of mobile robot. Sensors.

[18] Rybczak M., Popowniak N., Lazarowska A. (2024). A survey of machine learning approaches for mobile robot control. Robotics.

[19] Quang H.D., Tran T.L., Manh T.N., Manh C.N., Nhu T.N., Duy N.B. (2022). Design a nonlinear MPC controller for autonomous mobile robot navigation system based on ROS. Int. J. Mech. Eng. Robot. Res..

[20] Aro K., Urvina R., Deniz N.N., Menendez O., Iqbal J., Prado A. A nonlinear model predictive controller for trajectory planning of skid-steer mobile robots in agricultural environments. Proceedings of the IEEE Conference on AgriFood Electronics (CAFE).

[21] Fan Z., Wang L., Meng H., Yang C. (2025). RL-MPC-based anti-disturbance control method for pod-driven ship. Ocean. Eng..

[22] Outiligh A., Ayad H., El Kari A., Mjahed M., El Gmili N., Horváth E., Pozna C. (2024). An Improved IEHO Super-Twisting Sliding Mode Control Algorithm for Trajectory Tracking of a Mobile Robot. Stud. Inform. Control..

[23] Azegmout M., Mjahed M., El Kari A., Ayad H. (2023). New Meta-heuristic-Based Approach for Identification and Control of Stable and Unstable Systems. Int. J. Comput. Control..

[24] Fayti M., Mjahed M., Ayad H., El Kari A. (2023). Recent Metaheuristic-Based Optimization for System Modeling and PID Controllers Tuning. Stud. Inform. Control..

[25] Socha K., Blum C. (2006). Ant colony optimization. Metaheuristic Procedures for Training Neutral Networks.

[26] Ojha V.K., Abraham A., Snášel V. ACO for continuous function optimization: A performance analysis. Proceedings of the 14th International Conference on Intelligent Systems Design and Applications.

[27] Yun S., Zanetti R. (2021). Clustering methods for particle filters with Gaussian mixture models. IEEE Trans. Aerosp. Electron. Syst..

[28] Liu W., Wang Z., Yuan Y., Zeng N., Hone K., Liu X. (2019). A novel sigmoid-function-based adaptive weighted particle swarm optimizer. IEEE Trans. Cybern..

[29] Ait Dahmad H., Ayad H., Mousannif H., El Alaoui A. Fuzzy logic controller for 4WD/4WS autonomous agricultural robotic. Proceedings of the IEEE 3rd International Conference on Electronics, Control, Optimization and Computer Science (ICECOCS).

[30] Azizi M.R., Rastegarpanah A., Stolkin R. (2021). Motion planning and control of an omnidirectional mobile robot in dynamic environments. Robotics.

[31] Nfaileh N., Alipour K., Tarvirdizadeh B., Hadi A. (2022). Formation control of multiple wheeled mobile robots based on model predictive control. Robotica.

[32] Wang J., Liu Z., Chen H., Zhang Y., Zhang D., Peng C. (2023). Trajectory tracking control of a skid-steer mobile robot based on nonlinear model predictive control with a hydraulic motor velocity mapping. Appl. Sci..

[33] Xue Y., Wang X., Liu Y., Xue G. Real-time nonlinear model predictive control of unmanned surface vehicles for trajectory tracking and collision avoidance. Proceedings of the 7th International Conference on Mechatronics and Robotics Engineering (ICMRE).

[34] Andersson J.A.E., Gillis J., Horn G., Rawlings J.B., Diehl M. (2019). CasADi: A software framework for nonlinear optimization and optimal control. Math. Program. Comput..

[35] Haris M., Bhatti D.M.S., Nam H. (2024). A fast-convergent hyperbolic tangent PSO algorithm for UAVs path planning. IEEE Open J. Veh. Technol..

